# Effect of Arabinogalactans on Induction of White-Opaque Somatic Embryos of Avocado (*Persea americana* Mill.) cv. Duke-7

**DOI:** 10.3390/plants13010037

**Published:** 2023-12-21

**Authors:** C. L. Encina, A. Hamdi, R. Rodríguez-Arcos, A. Jiménez-Araujo, J. J. Regalado, R. Guillén-Bejarano

**Affiliations:** 1 Laboratorio de Cultivo de Tejidos y Biotecnología, Instituto de Hortofruticultura Subtropical y Mediterránea “La Mayora”, CSIC-UMA, Algarrobo-Costa, 29750 Málaga, Spain; 2 Grupo de Fitoquímicos y Calidad de Alimentos, Departmento Fitoquímica de Alimentos, Instituto de la Grasa (CSIC), Universidad Pablo de Olavide, Edificio 46 Ctra. de Utrera, km. 1, 41013 Sevilla, Spain; amelhamdi1988@yahoo.fr (A.H.); rrodri@ig.csic.es (R.R.-A.); araujo@ig.csic.es (A.J.-A.); rguillen@ig.csic.es (R.G.-B.); 3 Department of Biology and Geology, Agri-Food Campus of International Excellence (CeiA3) and Research Center CIAMBITAL, University of Almeria, 04120 Almeria, Spain; jjrg1984@ual.es

**Keywords:** AGP extraction, avocado cultivars, somatic embryogenesis, subtropical trees, WOSE induction

## Abstract

The development of somatic embryogenesis in avocado (*Persea americana* Mill.) has been hampered by different chronic problems. One such problem is the low level of induction of white-opaque somatic embryos (WOSEs) during the process of obtaining full avocado plants. We detected the induction of multiple WOSEs promoted after the placement of three or four small WOSEs over the embryogenic callus of Duke-7. Among the other possible chemical inductors of the Arabinogalactans (AGPs), we identified a family of extracellular plant proteoglycans implicated in many aspects of the in vitro induction of somatic embryos (SE). We extracted AGPs directly from embryogenic cultures of avocado. When the induction/proliferation medium of embryogenic avocado calli (MS-0.1 mg L^−1^ Picloram) was supplemented with 1–2 mg L^−1^ AGP, the induction rate of good-quality WOSEs from the embryogenic callus increased significantly (more than ten times that of the control without AGP) and this effect persisted for at least five subcultures after the initial treatment with AGP. AGP also modified the texture and quality of the callus. The effect of AGP extends to other cultivars and proliferation media. Our objectives were to improve the induction of WOSEs and study the effect of AGP in the somatic embryogenesis of avocado.

## 1. Introduction

The avocado, *Persea americana* Mill., is an important fruit crop worldwide, with a production of 8,685,672 t/year cultivated over 858,152 ha [1]. Mexico is the leading avocado producer of the tropical and subtropical countries. Spain is the 14th world producer with 116,770 t/year cultivated over 18,060 ha. The cultivar ‘Hass’ is the most commercially important cultivar of avocado [2]. The growth and production of avocado are affected by several diseases caused by pathogens such as *Phytophthora cinnamomi*, *Colletotrichum gloeosporioides* and some viroids [3,4]. The negative effects of these diseases on the avocado fruits are the greatest problem for avocado producers and can seriously affect international trade. At present, there are some selected genotypes with potential tolerance [5] to some of these diseases but this requires long-term work; all these diseases and the long juvenile periods of avocado delay the breeding programs in this species. Plant biotechnology and methods of the genetic edition and transformation of embryogenic cultures of avocado [6,7,8] appear as possible solutions to circumvent these and other problems, and somatic embryogenesis appears to be a suitable method for application in genetic studies in order to obtain avocado plants tolerant to diseases.

In addition to the development of micropropagation methods in avocado [9,10], different researchers have studied the somatic embryogenesis pathway and developed methods to regenerate full plants from somatic embryos (SE); however, these methods remain inefficient and quite dependent on the avocado genotype, resulting in low rates of regeneration [11,12,13,14,15,16,17,18]. Some success (2–37%) has been achieved in the regeneration of somatic embryos (SE) working with cultivars ‘Fuerte’ and ‘T362’, but the results are still dependent on the genotype and the rooting percentages were low [19]. Later, Witjaksono et al. [17] and Avenido et al. [20] also reported success (1.4–26.9%) in regenerating avocado plants from SEs, following a long and multiple-step process.

The induction of avocado SEs from embryogenic calli obtained from immature zygotic embryos promotes the formation of two types of somatic embryos; the first type is transparent SEs, which are unable to grow and further develop to a stage of maturity, and the second type corresponds to round white-opaque structures named WOSEs (e.g., embryos showing signs of maturity at very early stages). These embryos are capable of growing, undergoing further maturation and, finally, have a low conversion rate (1.4–26.9%) depending on the cultivar of avocado, giving rise to either shoots or roots and, on a few occasions, both types of organs (Encina, personal observation). Over the last few years of researching the somatic embryogenesis of avocado, we were hampered by different problems, the first being the poor generation and low quality of the WOSEs obtained, which severely limited the further development of regeneration methods from somatic embryos of avocado. The efficient regeneration of full plants from SEs is the second serious problem of this system [21], even further hindering the viability of genetic transformation using somatic embryos as a source. The problem of the induction of a high amount of good-quality somatic embryos (WOSE) in avocado was considered a secondary problem when most of the research was focused on the plant regeneration/conversion of somatic embryos [21,22]; this was a primary problem in cv. Duke-7, which presented severe difficulties in developing sufficient amounts of WOSEs. Even when the production of SEs is successful, the problem of low-quality somatic embryos remains as the rate of high-quality, bipolar embryos is extremely low (close to 2% or less) and genotype-dependent [13,23]. The use of glutamine and liquid media in regeneration seems to improve the ratio of regenerating SE embryos (58.3%), with 43.3% of regenerated SEs showing bipolar development [21]. Perán-Quesada et al. [24] indicated that the use of B5 major salts was essential in obtaining WOSEs, and sucrose (3%) plus gellan gum (0.68%) or coconut water (10–20%) also enhanced the recovery of WOSEs that were further grown on MS medium. Márquez-Martín et al. [25] also indicated the importance of the gelling agent in the development and maturation of the WOSEs of avocado, and they recommended the use of higher doses of agar (1%) than those considered as the standard. Guzmán-García et al. [26], using a 2D-DIGE and blind multivariate analysis, were able to separate different types of embryogenic cultures according to their capability to develop and maturate, indicating that different protein profiles can originate different morphogenetic behavior in avocado somatic embryogenesis. Palomo-Ríos et al. [22] analyzed the influence of semipermeable cellulose acetate membranes and culture media containing high levels of sucrose, along with coconut water, on the maturation and germination of somatic avocado embryos, increasing the number and quality of the WOSEs obtained.

In preliminary experiments working with an embryogenic line derived from a zygotic embryo of cv. Duke-7, we detected a bigger induction/proliferation of WOSEs when embryogenic calli were incubated together with some small WOSEs; this behavior was consistent (Encina, personal observation, 2020). AGPs are molecules shown to be involved in signaling in the somatic embryogenesis process [27,28,29,30,31,32,33,34,35,36,37]. Hence, AGPs could be the molecules involved in the interaction between embryogenic callus and WOSEs, resulting in a net improvement in WOSE proliferation. A preliminary chemical analysis of the media in which the cultures were grown supported this hypothesis [38].

AGPs are polymeric biomolecules consisting of arabinose and galactose monosaccharides. In plants, AGPs are frequently attached to proteins. AGP proteins are a family of extracellular plant proteoglycans that are rich in hydroxyproline/proline (Hyp/Pro), serine (Ser), alanine (Ala) and threonine (Thr) amino acids and characterized by an extensive glycosylation. AGPs contain a protein backbone of varying length (5–30 kDa) with N-terminal secretory peptide followed by AGP, fasciclin (FAS) domains and a C-terminal glycosylphosphatidylinositol (GPI) lipid anchor site [39]. Arabidopsis contains over 85 different AGPs [36,40], most of them containing a C-terminal GPI able to attach the extracellular AGPs to the plasma membrane and possibly act as signaling molecules [41]. AGPs are widely distributed in plants. AGPs are implicated in various aspects of plant growth and development, including root elongation, hormone responses, xylem differentiation, pollen tube growth and guidance, programmed cell death, cell expansion, salt tolerance, host–pathogen interactions, cellular signaling [42,43], sealing plant wounds and somatic embryogenesis in several plant species such as *Cyclamen persicum* [44], *Picea abies* [45] and *Daucus carota* [46,47]. In cotton [48], the specific AGPs produced by embryogenic calli were subsequently isolated and incorporated into tissue culture medium, which led to the promotion of cotton SEs. Additional studies revealed that the SE promoting activity resided in a hydrophobic fraction of AGPs. The functions of AGPs in plant growth and development processes rely heavily on the incredible diversity of their glycan and protein backbone residues [43]. In particular, it is the AG polysaccharides that are most likely to be involved in plant development [49].

Given that the improvement of regeneration methods and the plant conversion of avocado somatic embryos is the main goal of our current work, we investigated the effects of AGPs in the somatic embryogenesis process in avocado and focused on the generation of good-quality WOSEs in high quantities, combining chemical and biotechnological procedures.

## 2. Results

### 2.1. Preliminary WOSE Induction Bioassays

**Figure 1:**
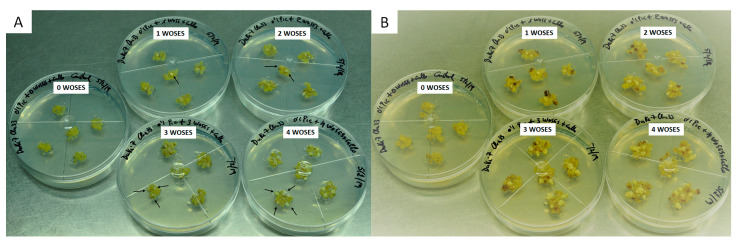
Induction of WOSEs by supplementing embryogenic callus explants of avocado cv. Duke-7 with (0, 1, 2, 3, 4 small WOSEs); isolated WOSE explants were used as control. Initiation of assay (**A**); end of assay after an incubation of 4 weeks (**B**). Arrows indicate the small WOSEs placed over the embryogenic calli.

In an avocado embryogenic callus obtained from immature zygotic embryos of cv. Duke-7, the addition of a growing number of small WOSEs resulted in a progressive increase in the induction and proliferation of new WOSEs in the standard culture medium, with the best results in cultures supplemented with three and four small WOSEs, generating, respectively, 2.8 and 3.2 new WOSEs per explant. For supplements of one and two small WOSEs, 1 and 1.4 new WOSEs were generated per explant, respectively. In the embryogenic callus not supplemented with WOSEs, the induction of new WOSEs was null. (Figure 1).

### 2.2. Isolation and Characterization of AGP

**Figure 2:**
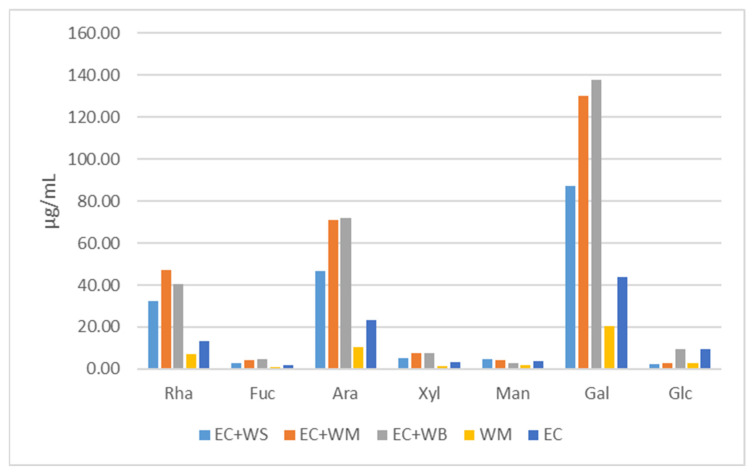
Composition of sugars in the dialyzed medium of the different samples. Abbreviations: (EC + WS)—embryogenic callus plus small-sized WOSEs; (EC + WM)—embryogenic callus plus medium-sized WOSEs; (EC + WB)—embryogenic callus plus large-sized WOSEs; (WM)—medium-sized WOSEs alone; (EC)—embryogenic callus alone; Rha—rhamnose; Gal—galactose; Ara—arabinose; Fuc—fucose; Xyl—xylose; Man—mannose; Glc—glucuronic acid.

These samples were incubated for a duration of 15 days in the liquid medium, as outlined in the Materials and Methods section. Subsequently, the culture medium was filtered through filter paper and dialyzed using a membrane with a molecular weight cutoff of 12,000. The sugar composition of the various samples is depicted in Figure 2. It is apparent that, in all cases, polysaccharides rich in galactose, arabinose and rhamnose accumulated in the dialysate. The maximum values for galactose (138, 130 and 88 µg mL^−1^) correspond to the samples consisting of EC plus WB, EC plus WM and EC plus WS; the higher values correspond to the large WOSEs. Something similar occurred for arabinose and rhamnose, where the greater accumulation of these polysaccharides corresponds to the samples involving EC plus different sizes of WOSEs. The rest of the compounds, fucose, xilose, mannose and glucuronic acid, were only accumulated in small amounts (<10 µg mL^−1^) for all samples tested. The combined trials involving embryogenic calli and WOSEs of different sizes exhibited a polysaccharide accumulation that was three to four times higher than the other samples.

The glycosyl composition of the polysaccharides (Table 1) is compatible with the presence of arabinogalactans: around 50% of galactose, 25% of arabinose and between 15 and 20% rhamnose. The other polysaccharides, xilose, mannose, fucose and glucuronic acid, were only present in a small percentage (from 2% to 10%).

The total sugars reached the maximum in the samples combining EC plus WOSEs of different sizes, with the highest being correlated with the biggest WOSEs (EC + WB > EC + WM > EC + WS), isolated WOSEs or EC.

The maximum amount of protein obtained from the culture media corresponded to EC and EC + WB (112.3 and 101.89 µg mL^−1^), with the rest of the samples showing significantly lower values of protein content. The polymeric material isolated from the medium in the three experiments that contained both EC and WOSEs was combined and freeze-dried. It was then subjected to two separate analyses: methylation analysis, to determine the types of bonds present in the carbohydrate part, and amino acid analysis, to determine the composition of the protein part.

The glycosyl linkage analysis (Table 2) clearly indicates the presence of arabinogalactans in the polysaccharides isolated from avocado cultures. AGPs are glycoproteins that contain approximately 2 to 9% protein. The carbohydrate component of AGPs consists of a linear chain of D-galactose molecules linked by β (1,3) bonds. Within this chain, some of the galactose units remain unsubstituted (3-Galp), while others are substituted at carbon 6 by β (1,6)-Gal side chains (3,6-Galp; 6-Gal). These side chains often carry terminal residues such as arabinose, fucose, rhamnose or glucuronic acid [50]. The glycosyl linkage analysis of the polysaccharides isolated from avocado cultures (as shown in Table 2) reveals that 3,6-Gal (30%) and 3-Gal (15%) are the main components of the polysaccharide. This indicates that approximately half of the main chain galactose units were substituted, providing valuable information about the structure of these polysaccharides originating from avocado cultures.

Regarding the protein portion, it is important to note that it represents approximately 23% of the total content when considering both sugars and proteins (Table 1). This suggests that some of the proteins in these samples may not be associated with arabinogalactan chains but may instead represent a fraction of proteins unrelated to them; clearly, in future studies, AGPs should be purified. In any case, Table 3 shows that the predominant amino acid is hydroxyproline (26%), with significant amounts of alanine (8%), serine (5%), threonine (3%) and glycine (7%). AGPs are generally classified into classic and non-classic categories. Classic AGPs have domains rich in hydroxyproline, alanine, serine, threonine and glycine, while non-classic AGPs are low in hydroxyproline and rich in cysteine or asparagine. Based on this, it appears that avocado AGPs belong to the classic group.

### 2.3. Isolation and Characterization of AGP WOSE Induction through AGP Supplementation

**Figure 3:**
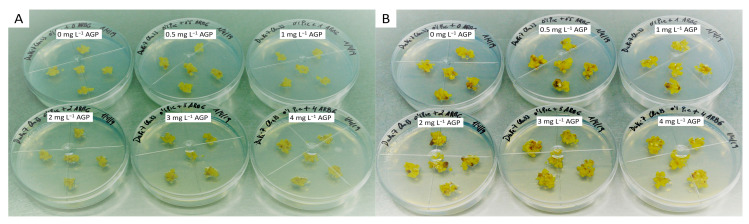
Test of WOSE induction by supplementing embryogenic callus explants of avocado cv. Duke-7 with different doses of AGP (0, 0.5, 1, 2, 3, 4 mg L^−1^). Initiation of assay (**A**); end of assay (**B**).

The results obtained from supplementing the proliferation medium with different doses of AGP added to the culture medium prior to autoclaving (Table 4, Figure 3) in order to incubate Duke-7 embryogenic callus showed that a high level of WOSE induction is obtained when the culture medium of the embryogenic callus is supplemented with 1–2 mg L^−1^ of AGP.

Our results also indicate that the quality of the embryogenic callus improved strongly when incubated in media supplemented with AGP; the callus shows a highly nodular texture, becoming harder and denser and even showing changes in color (from beige-brown to soft yellow-beige or translucent crystal). These changes in the color and texture of the callus can be correlated with a higher production of WOSE and a lesser proliferation of soft, friable, non-embryogenic calli. A higher quality of callus was obtained when the medium was supplemented with 1 mg L^−1^ of AGP, with lower rates of necrosis (14%) and a higher rate of good aspect calli (98%). The values obtained for 2 mg L^−1^ AGP are quite similar, and both doses of AGP can be considered suitable for further study. After several tests, we also detected that the method of adding AGP to the culture medium prior to autoclaving was suitable for WOSE induction; this method was adopted for further testing because it gave better results than the system whereby filter sterile solutions of AGP are added after media setting (Supplementary Materials Table S1) and allowed a reduction in the amount of AGP necessary for assaying.

**Figure 4:**
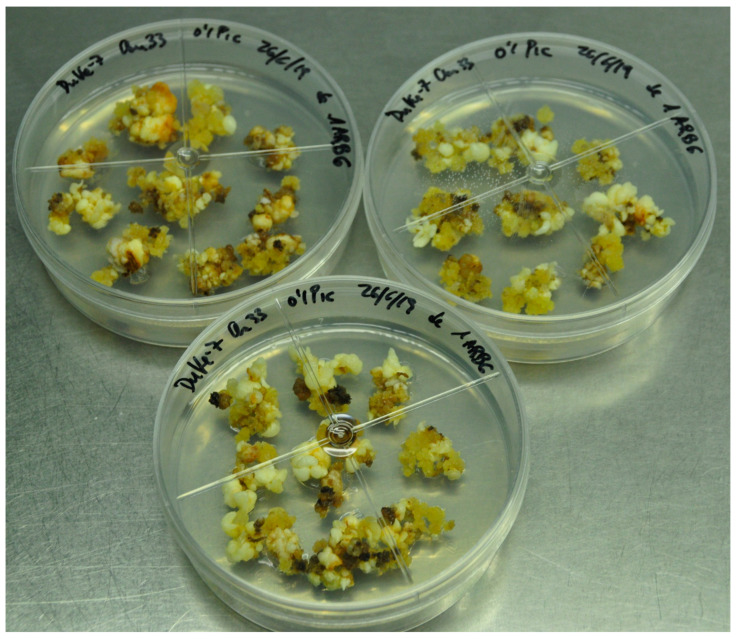
Persistence of the effect of supplementation with AGP (1 mg L^−1^) on WOSE proliferation and development after subculturing of explants to fresh medium without AGP.

When the embryogenic cultures were subcultured to standard fresh medium without AGP supplementation, the effect of induction and growth of WOSEs clearly persisted, at least for five subcultures (Figure 4).

### 2.4. WOSE Induction through AGP Plus One WOSE Supplementation

**Figure 5:**
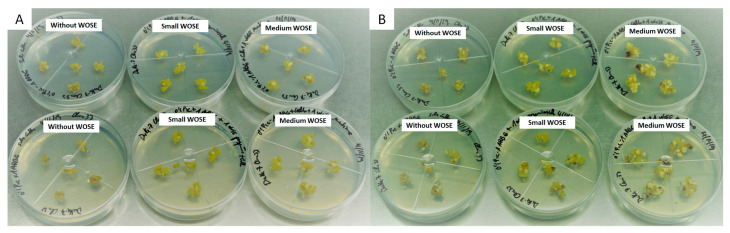
Induction of WOSEs by supplementing embryogenic callus explants of avocado cv. Duke-7 with 1 mg L^−1^ AGP plus one small-size WOSEs or one medium-size WOSE. Initiation of assay (**A**); end of assay after an incubation of 4 weeks (**B**).

As we can see in Table 5 and Figure 5, there were no significant differences between the average number of WOSEs induced by AGP supplemented with one WOSE or not, even if the value was higher for AGP without WOSE supplementation.

When a callus is supplemented with AGP and one medium-size WOSE, the number of WOSEs induced per explant is significantly higher with respect to the control AGP and the treatment with one small-size WOSE; this suggests some interaction between AGP and the presence of WOSEs. All this happened with a generally low percentage of necrosis in the culture, which points towards the generally high quality of the cultures in all treatments. There were no significant differences in the percentage of calli regenerating WOSEs and the percentage of explants with good aspect.

**Figure 6:**
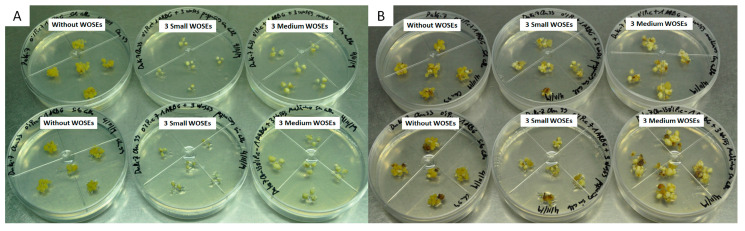
Effect of supplementation with 1 mg L^−1^ AGP over cv. Duke-7 explants consisting of 3 small-sized WOSEs and 3 medium-sized WOSEs, both without embryogenic calli. AGP was incorporated to the standard medium prior to autoclaving. Initiation of assay (**A**); end of assay after an incubation of 4 weeks (**B**).

The supplementation with AGP plus three WOSEs (Table 6) resulted in excellent quality cultures across all treatments and a significant increase in the number of WOSEs induced per explant in treatments involving the culture of three medium-sized WOSEs as explants. This result is significantly higher than the callus culture with AGP used as a control and the treatment involving the culture of three small-sized WOSEs (Figure 6).

The treatments involving the use of small-sized WOSEs as explants without embryogenic calli showed a general decrease in the number of explants with new WOSEs, and only a small amount of callus was able to grow from all the isolated WOSE explants. Due to the initial lack of callus in the primary explants used in the test, as expected, the differences in callus growth were significant. There were no significant differences in the percentage of callus-regenerating WOSEs, the percentage of necrosis or the percentage of explants with good aspect.

**Figure 7:**
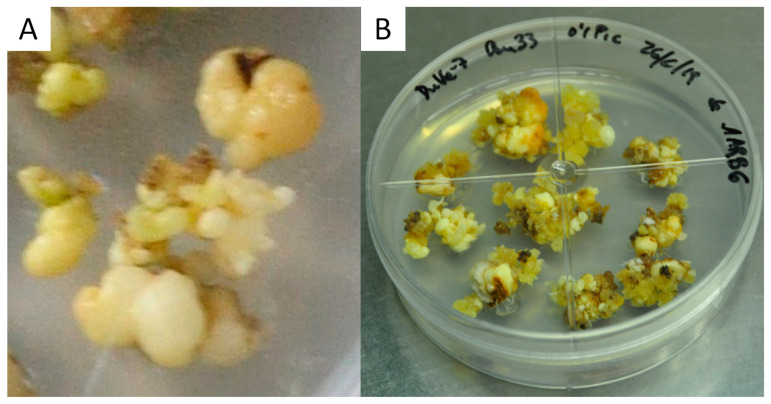
Secondary WOSE development in cultures of cv. Duke-7 incubated in medium MS + 0.1 picloram supplemented with 1 mg L^−1^ AGP on explants consisting of one medium-sized WOSE, without embryogenic callus (**A**) or with embryogenic callus (**B**), after 4 weeks of incubation.

Figure 7 shows, in detail, the induction and growth of secondary embryos in medium-sized WOSE explants of Duke-7 supplemented with 1 mg L^−1^ AGP.

## 3. Discussion

In oak [51], a fraction of the nodular embryogenic callus became a WOSE, even without AGP addition into the proliferation medium. In an avocado embryogenic callus under proliferation, a minimal fraction of the callus was also able to become a WOSE, but this extremely low percentage of WOSE generation (<0.5%) is useless for further purposes. However, it was feasible to induce higher rates of WOSEs when AGPs were included in the proliferation medium of the embryogenic callus of avocado. Apparently, the embryogenic behavior depends on the species and genotypes. Studies in carrot (*D. carota*), Norway spruce (*P. abies*), Persian cyclamen (*C. persicum*) and cotton (*G. hirsutum*) showed that AGPs derived from embryogenic culture medium, when added back to the culture medium, can promote SE in these species [44,45,52,53]. In cotton, the authors of reference [48] also compared the AGPs produced by embryogenic and non-embryogenic cotton cell cultures, detecting that both kinds of AGPs are different and produce a different effect on the process of somatic embryogenesis; different samples of AGPs were able to promote, inhibit or had no effects on the somatic embryogenesis of cotton. In our case, we extracted AGPs from embryogenic cultures and these are the kind of AGPs that were added back into our embryogenic cultures of cv. Duke-7.

In avocado cv. Duke-7, AGPs generated WOSEs from embryogenic calli when added to the induction/proliferation medium at concentrations ranging between 1 and 4 mg L^−1^. In agreement with our results, in cotton [48], ‘embryogenic’ AGPs also promoted somatic embryos (SE) at concentrations between 2 and 4 mg L^−1^. While there were no significant differences between the effects of embryogenic AGPs at the different concentrations tested, in avocado cv. Duke-7, the effects of AGP supplementation at concentrations between 1 and 2 mg L^−1^ were similar, but for the higher doses (3–4 mg L^−1^) tested, the induction of WOSEs was significantly less efficient. In avocado cv. Duke-7, when the embryogenic calli treated with AGP were transferred to fresh medium without AGP supplementation, the effect of generating new WOSEs persisted, at least for five consecutive subcultures. This effect faded gradually after 10 subcultures. Examining our results combining AGP plus WOSEs, it is possible that the high number of WOSEs growing in vitro increased the amount of AGPs released to the medium and that the system can generate the necessary AGP by itself, maintaining the generation rate of WOSEs without any external supplementation.

Our experiments attempting to verify the possible effects of AGP supplementation on differently sized WOSEs revealed that AGP was able to induce the generation of new WOSEs in the medium-sized WOSEs tested, apparently inducing secondary embryogenesis and significantly increasing the number of WOSEs induced per explant (Figure 7); however, secondary embryogenesis was not induced on small WOSEs. AGP induces de novo WOSEs from embryogenic calli in treatments without WOSEs or including one small WOSE per explant (Table 5). These differences could be due to the higher level of reserves available in the bigger WOSEs, allowing them to develop secondary WOSEs. Figure 7 shows, in detail, the induction and growth of secondary embryos in medium-sized WOSE explants of Duke-7 supplemented with 1 mg L^−1^ AGP.

Our results also show that AGP supplementation improved the quality of the embryogenic callus, which became denser and more nodular, and even showed changes in color from beige-brown to soft yellow-beige or translucent crystal; this change in callus texture could be the first step in obtaining a higher induction of WOSEs from this improved embryogenic callus. These results are consistent with the observations of Mallon et al. [54] and Shu et al. [55] in *Quercus* sp. and *Musa* sp. indicating that non-embryogenic cells can be stimulated to undergo embryogenesis when treated with purified AGPs. In avocado, it appears that something similar occurs whereby non-embryogenic cells are converted into embryogenic cells, improving the quality of the embryogenic calli of avocado.

An analysis of the results obtained after testing the effect of AGP over isolated groups of small- and medium-sized WOSEs (without any callus), together with the assays involving AGP supplementation plus supplementation with WOSE in embryogenic callus explants, suggest that the AGP (a) acts in promoting de novo embryogenesis in the explants of embryogenic calli; (b) acts in promoting secondary embryogenesis in WOSE explants when there is no callus available; and (c) acts in promoting secondary embryogenesis and de novo embryogenesis together when calli plus WOSEs are present together as explants. Apparently, the secondary embryogenesis reached higher levels of medium-sized WOSEs than small-sized WOSEs. (Figure 7). The results obtained for embryogenic calli induced from immature zygotic embryos of cv. Duke-7 in medium MS plus 0.1 mg L^−1^ plus AGP during the phase of proliferation were also obtained when the embryogenic callus was induced and maintained under proliferation in another culture medium MS plus 2,4-D plus TDZ plus AGP. Through applying AGPs to proliferating embryogenic cultures obtained from immature zygotic embryos from other cultivars of avocado (cv. Anaheim, cv. Reed, cv. Y381/PAC1), we also obtained an improvement in the quality of embryogenic calli and the development of WOSEs from these avocado materials. Apparently, the capability of AGP to generate embryogenic changes and WOSEs can be extended to different cellular lines of avocado and culture conditions.

At present, we are trying to establish whether it is more efficient to produce WOSEs starting from isolated WOSEs as explants or starting from embryogenic calli, and how to settle the quality of the WOSEs obtained with each type of material, which is very important for the final step of regeneration and plant recovery in avocado somatic embryo cultures.

## 4. Materials and Methods

### 4.1. In Vitro Somatic Embryogenesis

Embryogenic cultures of avocado (*Persea americana* Mill.), cultivar Duke-7, were initiated following the protocols of Witjaksono and Litz [15]. Using immature zygotic embryos as explants, embryogenic callus cultures were induced in Petri dishes containing MS medium (macro and micro salts) [56], supplemented with 0.1 mg L^−1^ picloram and, in (mg L^−1^): thiamine-HCl (100), pyridoxine-HCl (50), nicotinic acid (50), glycine (200), i-inositol (100), sucrose (3%) and Agar SIGMA A-1296 (Sigma Aldrich, St. Louis, MO, USA) (6%). After initiation, cultures were maintained on the same medium and incubated in darkness at 17 ± 2 °C. All media were adjusted to pH 5.7 prior to autoclaving at 1.1 kg cm^−2^ and 121 °C for 20 min and Petri dishes were sealed with parafilm.

### 4.2. Somatic Embryogenesis Bioassays

The medium for these assays consisted of MS macro and micro salts supplemented with 0.1 mg L^−1^ picloram, as indicated above. The initial experiments involved supplementation of the embryogenic callus cultures with WOSEs; in these assays, small WOSEs (0.5 to 1.5 mm diameter in size) were placed over the callus surface. Every callus was supplemented with a different number of WOSEs (0, 1, 2, 3, 4 per callus). The calli plus the WOSEs were incubated in the standard medium indicated above. Medium-sized WOSEs corresponded to sizes 2–4 mm in diameter. Data on the callus size (0–5): 0—lack of callus; 1—0.4 cm diameter (Ø); 2—0.8 cm Ø; 3—1.2 cm Ø; 4—1.6 cm Ø; 5–2 cm Ø.

### 4.3. Extraction of AGPs

Five distinct avocado samples were prepared, comprising embryogenic callus plus small-sized WOSEs (EC + WS), embryogenic callus plus medium-sized WOSEs (EC + WM), embryogenic callus plus large-sized WOSEs (EC + WB), medium-sized WOSEs alone (WM) and embryogenic callus alone (EC). These were incubated for 15 days in liquid medium (MS salts plus 0.1 mg L^−1^ picloram) in an orbital shaker at 100 rpm; during this time, AGPs were released into the culture medium. The culture medium (100 mL) was filtered through filter paper and subsequently dialyzed using cellulose membranes with a molecular cut-off of 12,000 Da. This dialysis process was carried out against 10 L of distilled water for 12 h. Following this, the distilled water was replaced, and dialysis was continued for an additional 12 h. The carbohydrate composition in the dialysate was analyzed by GC according to the alditol acetates method, as previously described [57]. Methylation analysis was carried out according to the method of Ciucanu and Kerek [58]. Protein was quantified on the basis of the dye-binding method of Bradford using bovine serum albumin (BSA) as standard [59]. Amino acids were determined after acid hydrolysis and derivatization with ethoxy methylene malonate by HPLC-DAD [60]. The AGP used in the supplementation assays was a dry powder derived from the dialyzed and lyophilized extract. According to chemical analysis, the major components of this powder are arabinogalactans and their associated proteins.

### 4.4. AGP Supplementation Assays

For preliminary assays involving AGP supplementation, regular pieces of embryogenic callus were transferred to fresh control medium. The different concentrations of AGP (0, 0.05. 0.1, 0.5, 1, 2, 3 mg L^−1^) were incorporated as filter-sterilized solutions just prior to the media setting (<60 °C). As additional controls, we prepared some plates including an embryogenic callus with one small WOSE with or without AGP. These treatments consisted of four incubation weeks of three Petri dishes containing five round pieces (5 mm diameter) of embryogenic callus.

AGP was always added directly to the medium prior to autoclaving. The callus explants, 5 mm in diameter, were paced in Petri dishes, each containing 5 round pieces (5 mm diameter) and incubated for 4 weeks; after this period, the number of explants that had developed WOSEs was recorded. All treatments used 12 Petri dishes, with a total of 60 explants, distributed in three independent experiments.

### 4.5. AGP Plus WOSE Supplementation Assays

We also studied the effect of 1 mg L^−1^ AGP supplementation on the embryogenic callus plus one small-sized WOSE (1 mm diameter) or a medium-sized WOSE (3 mm diameter) added on the embryogenic callus (Table 5, Figure 5). Additionally, we tested the effect of 1 mg L^−1^ AGP supplementation on explants consisting of 3 small WOSE and on explants consisting of 3 medium WOSEs without embryogenic calli (Table 6, Figure 6).

### 4.6. Statistical Analysis

All the data were analyzed using the SPSS software package (version 22.0; SPPS INC., Chicago, IL, USA). The variables percentage of callus with formation of WOSEs and percentage of explants with good aspect were analyzed by generalized linear models using Logit as the link function and Binomial as the probability distribution. Pairwise comparisons among groups were performed using Fisher’s least significant difference (LSD) test. While linear variables with a normal distribution (callus size, number of WOSEs obtained in each callus and callus with necrosis) were analyzed via one-way ANOVA, we used a HSD Tukey test in the post hoc analysis for comparisons among groups.

## 5. Conclusions

The process of WOSEs generation in embryogenic calli proliferated from immature zygotic embryos of cv. Duke-7 was clearly improved with the addition of AGPs extracted from mixtures of embryogenic callus and WOSEs to the proliferation medium; the effect of this supplementation persisted amongst the following subcultures of the SE cultures treated with AGPs without further supplementation with additional AGP. Doses between 1 and 2 mg L^−1^ appear to be optimal for the induction and growth of WOSEs in the cultivar Duke-7 of avocado. AGP extracts can be successfully applied to induce WOSEs in other genotypes of avocado and other culture media. An analysis of our results suggests that the AGPs (a) act in promoting de novo embryogenesis when explants consist of an embryogenic callus; (b) act in promoting secondary embryogenesis in WOSEs explants when there is no callus available; and (c) act in promoting secondary embryogenesis and de novo embryogenesis together when a callus plus WOSEs are present together as an explant.

## Figures and Tables

**Table 1:** Glycosyl composition (%), total sugars and proteins of the polysaccharides isolated from the culture media. Values are means of three replicates ± SD.

	Glycosyl Composition (%)							Total Sugars (µg mL^−1^)	Proteins (Bradford) (µg mL^−1^)
	Rha	Fuc	Ara	Xyl	Man	Gal	Glc		
EC + WS	18	2	26	3	3	48	1	181.5 ± 0.8 a	38.6 ± 9.0 a
EC + WM	18	2	27	3	2	49	1	266.3 ± 1.5 b	72.5 ± 6.0 b
EC + WB	15	2	26	3	1	50	3	274.6 ± 0.7 b	101.9 ± 12.2 c
WM	16	2	23	3	4	46	7	44.9 ± 1.5 c	44.6 ± 8.0 a
EC	14	2	23	3	4	45	10	98.4 ± 5.3 d	112.3 ± 13.0 c

Different letters in the same column indicate significant differences (*p* < 0.05). Abbreviations: (EC + WS)—embryogenic callus plus small-sized WOSEs; (EC + WM)—embryogenic callus plus medium-sized WOSEs; (EC + WB)—embryogenic callus plus large-sized WOSEs; (WM)—medium-sized WOSEs alone; (EC)—embryogenic callus alone; Rha—rhamnose; Gal—galactose; Ara—arabinose; Fuc—fucose; Xyl—xylose; Man—mannose; Glc—glucuronic acid.

**Table 2:** Glycosyl linkage analysis of polysaccharides isolated from the growing media.

PMAA Derivative	Deduced Linkage	%mol
1,5-Di-O-acetyl-1-deuterio-6-deoxy-2,3,4-tri-O-methyl-L-mannitol	T-Rham	10
1,4-Di-O-acetyl-1-deuterio-2,3,5-tri-O-methyl-D-arabinitol	T-Araf	18
1,5-Di-O-acetyl-1-deuterio-2,3,4-tri-O-methyl-D-arabinitol	T-Arap	3
1,3,4-Tri-O-acetyl-1-deuterio-2,5-di-O-methyl-D-arabinitol	3-Araf	6
1,4,5-Tri-O-acetyl-1-deuterio-2,3-di-O-methyl-D-arabinitol	4-Arap	3
1,3,5-Tri-O-acetyl-1-deuterio-2,4,6-tri-O-methyl-D-galactitol	3-Galp	15
1,5,6-Tri-O-acetyl-1-deuterio-2,3,4-tri-O-methyl-D-galactitol	6-Galp	9
1,4,5,6-Tetra-O-acetyl-1-deuterio-2,3-di-O-methyl-D-galactitol	4,6-Galp	4
1,3,5,6-Tetra-O-acetyl-1-deuterio-2,4-di-O-methyl-D-galactitol	3,6-Galp	30
1,3,4,5,6-Penta-O-acetyl-1-deuterio-2-O-methyl-D-galactitol	3,4,6-Galp	3

**Table 3:** Amino acid composition of AGPs proteins.

Amino Acid	Concentration (µg mg^−1^)	%
Aspartic A.	22.4 ± 1.2 a	11
Glutamic A.	21.9 ± 2.1 a	11
Hydroxyproline	52.9 ± 2.4 b	26
Serine	10.0 ± 0.8 c	5
Glycine	14.8 ± 1.7 d	7
Threonine	6.4 ± 0.5 e	3
Arginine	10.7 ± 1.8 c	5
Alanine	15.3 ± 1.3 d	8
Valine	2.9 ± 0.8 f	1
Methionine	19.7 ± 0.4 a	10
Cystine	2.8 ± 0.4 f	1
Leucine	8.6 ± 0.7 e	4
Phenylalanine	5.3 ± 0.6 e	3
Lysine	8.1 ± 0.5 e	4
Total	201.6 ± 13.2	100

Values are means of three replicates ± SD different letters in the same column indicate significant differences (*p* < 0.05).

**Table 4:** Effect of supplementation of different doses of AGP (0, 0.5, 1, 2, 3, 4 mg L^−1^) on embryogenic calli derived from a zygotic embryo of cv. Duke-7 incorporated into the standard medium prior to autoclaving.

Concentration of AGP (mg L^−1^)	Callus Size (0–5)	No. of New WOSEs per Callus	Callus with WOSEs %	Necrosis %	Good Aspect Explants %
0	2.3 ± 0.6 b	0.16 ± 0.1 c	16 ± 5 c	32 ± 2 a	92 ± 4 ab
0.5	1.9 ± 0.7 c	0.22 ± 0.1 c	22 ± 6 c	18 ± 2 c	96 ± 3 a
1	2.9 ± 0.5 a	1.70 ± 0.2 a	84 ± 5 a	14 ± 1 c	98 ± 2 b
2	2.1 ± 0.5 bc	1.52 ± 0.2 ab	80 ± 6 a	23 ± 1 b	96 ± 3 a
3	2.9 ± 0.5 a	1.04 ± 0.1 b	68 ± 7 ab	29 ± 1 ab	96 ± 3 a
4	2.8 ± 0.5 a	1.06 ± 0.2 b	58 ± 7 b	33 ± 1 a	82 ± 5 b

Data on callus size, no. of new WOSEs and no. of callus regenerating WOSEs were recorded after an incubation of 4 weeks. Different letters indicate groups that were significantly different at α= 0.05 using LSD test, in percentages of callus regenerating WOSEs and percentages of explants with good aspects, and by HSD-Tukey at α = 0.05 in callus size (0–5), no. of new WOSEs per callus and percentage of necrosis.

**Table 5:** Effect of supplementation with 1 mg L^−1^ AGP plus one small-size WOSE/explant, or plus one medium-size WOSE/explant, on embryogenic callus of cv Duke-7 incorporated into standard medium prior to autoclaving.

No. of WOSEs Added Per Callus Explant	Callus Size (0–5)	No. of New WOSEs Per Explant	Callus with WOSEs %	Necrosis %	Good Aspect Explants %
0 WOSEs	2.8 ± 0.7 a	1.44 ± 0.1 ab	84 ± 5 a	19 ± 1 a	96 ± 3 a
1 small WOSE	1.8 ± 0.5 b	1.42 ± 0.2 b	70 ± 7 a	11 ± 0 b	100 ± 0 a
1 medium WOSE	1.8 ± 0.5 b	2.02 ± 0.2 a	72 ± 6 a	20 ± 0 a	100 ± 0 a

Data on callus size (0–5), no. of new WOSEs per callus, percentage of callus-regenerating WOSEs and percentage of explants with good aspect were recorded after an incubation of 4 weeks. Different letters indicate groups that were significantly different by HSD-Tukey at α= 0.05 in terms of callus size (0–5), no. of new WOSEs per callus and percentage of necrosis.

**Table 6:** Effect of supplementation with 1 mg L^−1^ AGP over cv Duke-7 callus explants consisting of 3 small-sized WOSEs and 3 medium-sized WOSEs. AGP was incorporated to the standard medium prior to autoclaving.

No. of WOSEs Added Per Explant	Callus Size (0–5)	No. of New WOSEs Per Explant	Callus with WOSEs %	Necrosis %	Good Aspect Explants %
Callus + 0 WOSEs	2.8 ± 0.7 a	1.4 ± 0.1 b	84 ± 5 a	19 ± 1 a	96 ± 3 a
3 small WOSEs	1.2 ± 0.4 c	1.7 ± 0.2 b	74 ± 6 a	22 ± 1 a	94 ± 3 a
3 medium WOSEs	1.8 ± 0.4 b	2.4 ± 0.3 a	72 ± 6 a	21 ± 1 a	96 ± 3 a

Data on callus size (0–5), no. of new WOSEs per callus, percentage of callus regenerating WOSEs, necrosis rate and percentage of explants with good aspect were recorded after an incubation of 4 weeks. Different letters indicate groups that were significantly different by HSD-Tukey at α= 0.05 in callus size (0–5) and no. of new WOSEs per callus.

## Data Availability

Data are available within the article and Supplementary Materials.

## Supplementary Materials

The following supporting information can be downloaded at: https://www.mdpi.com/article/10.3390/plants13010037/s1.
