# Supplementary material for: Effect of Arabinogalactans on Induction of White-Opaque Somatic Embryos of Avocado (Persea americana Mill.) cv. Duke-7
Source: Plants (Basel). 2023 Dec 21;13(1):37. doi: 10.3390/plants13010037 (PMC10780364; doi:10.3390/plants13010037)
Supplement: Supplementary file 1 [file plants-13-00037-s001.zip › plants-2764953-supplementary.pdf]

Table S1. Effect of different doses of AGP (0, 0.05. 0.1, 0.5, 1, 2**,** 3 mg l^{-1}) incorporated aseptically to explants with a micropipette as filter-sterilized solutions just prior to the media setting.

AGP mg l^{-1}	Callus Size (0-5)	No. of new WOSEs per Callus	Callus with WOSEs %	Necrosis %	Good Aspect Explants %
0	3.4 ± 0.6 ^{bc}	0.06 ± 0.1 ^{b}	7 ± 3 ^{c}	22 ± 4 ^{a}	73 ± 8 ^{a}
0.05	3.7 ± 0.6 ^{b}	0.03 ± 0.0 ^{b}	3 ± 3 ^{c}	20 ± 3 ^{a}	77 ± 8 ^{a}
0.1	4.6 ± 0.5 ^{d}	0.03 ± 0.0 ^{b}	3 ± 3 ^{c}	32 ± 4 ^{a}	47 ± 9 ^{b}
0.5	2.8 ± 0.8 ^{d}	0.27 ± 0.1 ^{b}	20 ± 7 ^{bc}	27 ± 4 ^{a}	73 ± 8 ^{a}
1	2.9 ± 0.7 ^{cd}	0.87 ± 0.1 ^{a}	63 ± 9 ^{a}	31 ± 5 ^{a}	73 ± 8 ^{a}
2	2.7 ± 0.4 ^{d}	0.50 ± 0.1 ^{ab}	40 ± 9 ^{ab}	33 ± 4 ^{a}	47 ± 9 ^{b}
3	3.8 ± 0.7 ^{b}	0.90 ± 0.3 ^{a}	33 ± 9 ^{c}	32 ± 4 ^{a}	47 ± 9 ^{b}

Data of callus size (0-5), No. of new WOSEs per callus, percentage of callus regenerating WOSEs and percentage of explants with good aspect, were recorded after an incubation of 4 weeks. Different letters indicate groups that were significantly different by LSD at α= 0.05, in percentage of callus regenerating WOSEs and percentage of explants with good aspect, and by HSD-Tukey at α= 0.05, in callus size (0-5) and No. of new WOSEs per callus. There are no significant differences in percentage of necrosis.
